# Development of a Low-Power IoMT Portable Pillbox for Medication Adherence Improvement and Remote Treatment Adjustment

**DOI:** 10.3390/s22155818

**Published:** 2022-08-04

**Authors:** Dimitrios Karagiannis, Konstantinos Mitsis, Konstantina S. Nikita

**Affiliations:** Biomedical Simulations and Imaging Lab, School of Electrical and Computer Engineering, National Technical University of Athens, 15780 Athens, Greece

**Keywords:** 3D printing, drug interactions, image recognition, internet of medical things, IoMT, low-power device, medication adherence, personalized medicine, pillbox

## Abstract

Patients usually deviate from prescribed medication schedules and show reduced adherence. Even when the adherence is sufficient, there are conditions where the medication schedule should be modified. Crucial drug–drug, food–drug, and supplement–drug interactions can lead to treatment failure. We present the development of an internet of medical things (IoMT) platform to improve medication adherence and enable remote treatment modifications. Based on photos of food and supplements provided by the patient, using a camera integrated to a portable 3D-printed low-power pillbox, dangerous interactions with treatment medicines can be detected and prevented. We compare the medication adherence of 14 participants following a complex medication schedule using a functional prototype that automatically receives remote adjustments, to a dummy pillbox where the adjustments are sent with text messages. The system usability scale (SUS) score was 86.79, which denotes excellent user acceptance. Total errors (wrong/no pill) between the functional prototype and the dummy pillbox did not demonstrate any statistically significant difference (*p* = 0.57), but the total delay of the intake time was higher (*p* = 0.03) during dummy pillbox use. Thus, the proposed low-cost IoMT pillbox improves medication adherence even with a complex regimen while supporting remote dose adjustment.

## 1. Introduction

Even correct medication can lead to decreased efficacy when patients do not adhere to their prescribed schedule. The World Health Organization states that only 50% of patients with chronic diseases in developed countries adhere to their prescribed treatment [1]. Although medication adherence improvement increases treatment costs for some diseases, this leads to decreased hospitalizations and emergency events, thus lowering overall costs [2]. Moreover, limited medication adherence can lead to adverse results like cardiovascular mortality of patients with coronary artery disease (CAD) [3]. Adverse drug events (ADEs) and medication errors that are 71% caused in the prescribing stage cause significant health costs, while most of them are preventable [4].

Various devices have been identified in recent review studies [5] to monitor and improve medication adherence. These devices incorporate different mechanisms to notify patients about the time of their dose and further monitor whether they adhere to their prescribed schedule. MedTracker is a portable pillbox that offers continuous adherence monitoring but provides a rather limited capacity [6]. eDosette uses an image sensor to recognize changes in a patient’s blister packages or dosettes, thus monitoring medication adherence, but cannot be transferred outside of a patient’s house because of its size [7]. A wearable wristband can notify patient at the correct time to receive medication from a 3D-printed seven-compartment pillbox while a patient’s wristband gesture and pillbox sensors are used to indicate medication intake [8]. Moreover, a pillbox using a motor that automatically opens the pill door and notifies through SMS has been suggested [9]. A Wi-Fi programmable multi-chamber pillbox has been also proposed, where the pills are prepared per dose in each chamber, an LED provides alert signals to the patient, and a light sensor is used to monitor adherence [10]. Another approach employs two-way Wi-Fi communication between the pillbox and a webpage that enables pill intake monitoring with sensors, while remote relatives can send text or voice messages back to the device to help the patient [11]. A low-power pillbox with IoT capabilities has been described that besides notifying about the medication intake can manage expired stored medicines [12]. Other platforms integrate bio-signals acquisition to measure ECG and body temperature in addition to medication adherence services [13]. Moreover, IoT-cyber physical systems (IoT-CPS) with artificial intelligence have been suggested to predict diseases [14]. Hero [15] is a commercial device that provides internet functionalities, automatically dispenses pill combinations, and can hold up to 10 different types of medications, at the expense of being bulky and not portable. Pria [16], another commercial product, offers advanced functionalities, including camera-based facial recognition of the user, and provides adherence data and capacity up to 28 doses, but it is not intended for portable use despite its embedded battery.

Although adherence is important for successful pharmacotherapy, other factors, such as a patient’s eating habits, which are usually unknown to the prescribing physician, can lead to unexpected results. Many drugs may interact not only with other drugs [17], but also with specific food or supplement ingredients. These interactions can cause treatment failure because of reduced or increased drug bioavailability [18,19,20]. During prescription, the physician can check other medications taken by the patient and identify potentially harmful interactions. Thus, drug–drug interactions can be easily identified and prevented. On the contrary, food–drug and supplement–drug interactions may occur during a patient’s everyday routine and are difficult to be monitored and prevented. Although the medication adherence devices proposed in the literature can successfully monitor or improve adherence, they are not able to investigate interactions with a patient’s food or supplement intake.

Furthermore, as they require preparation in advance doses with combinations of medicines, they do not allow complex regimen adjustments, like postponing or adding a specific pill to prevent drug interactions or controlling other biomarkers (e.g., low blood sugar or elevated blood pressure) that arise during the day. Frequently, these devices are too bulky for the patients to carry [15], or require a constant energy supply, thus limiting device portability. Battery optimization on a smartphone can increase compliance to remote healthcare systems, as there is no need for frequent charging [21]. Low-power design can lead to improved compliance compared with similar high-power-demanding devices. Simplistic solutions based on custom packages, where the pill combination for each dose is in a separate container [22], can improve adherence without an electronic device but they require a technologically advanced network of pharmacy stores around the patient’s location that can prepare and distribute custom packages. Although such solutions can be employed in certain urban areas, they remain economically unfeasible in sparsely populated or remote areas. Internet connectivity incorporated in recent medication adherence devices focuses on remote adherence monitoring and does not allow automated interventions with remote dose adjustments.

In this paper, we present the design of a pillbox with internet access to enable continuous, low-power, and autonomous device interaction with an internet of medical things (IoMT) platform. Object recognition techniques are integrated to perform such complex health-related tasks as the detection of patient eating habits and the prevention of dangerous interactions with their medication. Object detection and identification has been developed in recent years and is applied in many fields [23]. Moreover, computer vision techniques can provide important data in health care where facial images can be used for BMI prediction and identification of patient obesity or malnutrition conditions [24]. Unlike previous pillbox designs that focused on adherence detection techniques that accurately compute pill intake, we propose an IoMT approach capable of combining pharmacotherapy monitoring with new features that integrate extra functionalities, such as pill-storage condition monitoring, food–drug and supplement–drug interactions identification, and remote adjustments of complex medication schedules, along with improved device portability. From an end-user perspective, the proposed platform consists of a pillbox and a web application that allows improved monitoring and medication adherence. The pillbox is battery powered with a low-power consumption design to ensure portability. The platform aims to improve personalized pharmacotherapy through the use of an infrastructure based on affordable technologies, including 3D printing, modern microcontrollers, and powerful APIs. In addition, it offers an unobtrusive way to obtain critical information regarding patients’ daily routines, such as eating habits and pill storage conditions. A carefully designed two-part experimental procedure has been conducted to assess the energy consumption associated with the use of the proposed platform, along with issues related with user acceptance and the potential impact on medication adherence.

The rest of the paper is organized as follows. The architecture and functionalities of the proposed IoMT platform are presented in Section 2, along with the design of the experimental study aiming at evaluating power consumption, medication adherence, and user acceptability. The study’s results are presented in Section 3 and discussed in Section 4.

## 2. Materials and Methods

### 2.1. Architecture of the Proposed IoMT Platform

**Figure 1:**
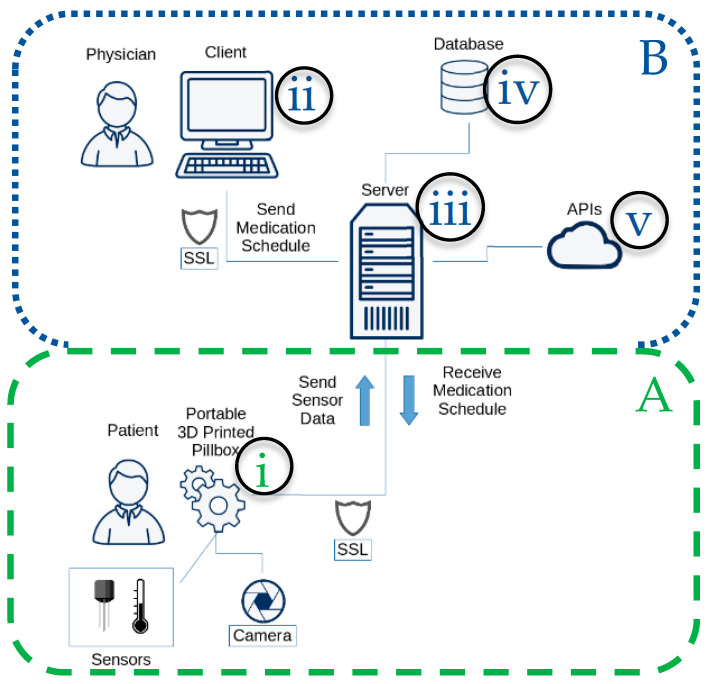
IoMT platform overview: Space A supports the interaction with the patient through the pillbox functionalities, while Space B includes the platform’s backend (server, database, cloud services) and frontend supporting a physician’s interaction through the web app.

The developed IoMT platform consists of: (i) a 3D printed pillbox, (ii) a web application, (iii) a remote server, (iv) a database, and (v) third party APIs (Figure 1). The proposed architecture aims at improving medication adherence and detecting interactions of the current therapy medicines with newly added medicines, food, and supplements consumed by the patient.

The pillbox is a 3D-printed enclosure with a folded small-sized design that provides a high pill storage capacity [25]. Through appropriate embedded sensors, the pillbox monitors environmental temperature and humidity. Sound and light signals notify the patient about the correct pill to be taken at the appropriate time. An integrated camera allows the patient to take a photo of food, drink, or supplement that is then processed by the cloud services to identify potential food–drug and supplement–drug interactions. Through the web application, physicians or pharmacists can check drug–drug interactions while they prescribe new medicines to their patients. The updated medication schedule is then securely transferred to the remote server.

The remote server undertakes secure communication (SSL/TLS) with the pillbox [26] and the web application and implements the interaction with the database. All information related to the patients and their medication schedules, along with sensor measurements, drugs, and their known interactions, as well as recorded occurred interactions, are stored in the database. To check drug–drug interactions, the National Medicine Library Interaction API has been used [27,28,29,30]. The Google Cloud Vision API [31,32] has been used for image recognition to provide data used for food–drug and supplement–drug interactions detection.

### 2.2. Patient Interaction Space

#### 2.2.1. 3D Printed Folding Enclosure and Electronic Components

**Figure 2:**
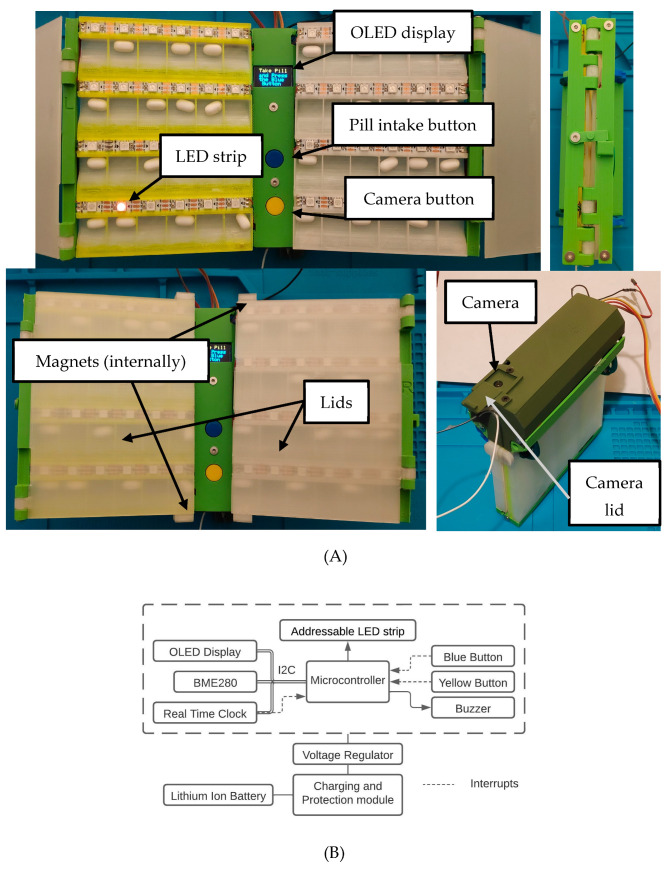
(**A**) 3D-printed pillbox: top-left: unfolded structure, top-right: completely folded structure, bottom-left: folded lids, bottom-right: rear view. (**B**) Simplified pillbox hardware architecture. OLED display, BME280, and real-time clock communicate with the microcontroller using the I2C protocol. The blue and yellow buttons and the real-time clock operate as interrupts. (Figure 2B created in Lucidchart. Available: www.lucidchart.com).

The pillbox has a unique folding structure that enables a small size and a storage capacity for up to 48 individually controlled pills (Figure 2A). The pillbox case is 3D printed and can be easily reproduced without expensive industrial equipment. The two lids hold pills in place during pillbox opening and both panels close to protect the display and the buttons, reducing device size. The use of an embedded microcontroller, which is programmed to operate in low power, along with the hand-sized structure, enhance portability and allow patients to carry the pillbox during their outdoor activities.

The electronic components, placed on a custom-designed PCB (Supplementary Figure S1) inside the 3D-printed enclosure, include (Figure 2B): (i) a microcontroller (ESP32-CAM) that enables low-power operation while not used, controls the other components, and communicates with the remote server, and a camera embedded in the microcontroller enables the patient to take photos of food and supplements to check for interactions; (ii) a real-time clock (RTC—DS3231) that continuously preserves time even when the microcontroller is reset or the battery voltage is low; (iii) a buzzer that notifies the patient with a sound signal at the correct dose time; (iv) an OLED display that interacts with the patient providing appropriate instructions; (v) an addressable LED strip that indicates the correct pill for intake—every LED is individually controlled from the microcontroller, thus providing a way to modify proposed doses in real-time as in the case of an identified food-drug or supplement-drug interaction and indicate any possible pill combination without the need to rearrange them; (vi) a BME280 sensor that measures temperature and humidity; these measurements are crucial for pills storage conditions and the patient’s environment conditions; and (vii) a module that recharges the Li-ion battery without being removed from the device. The pillbox’s low-cost components and 3D-printed structure make its cost comparable to other available pillboxes that provide similar or even reduced functionalities [33]. To maintain low-power consumption, the device buttons and the RTC alarms operate as interrupts while the microcontroller is in deep-sleep state. The pillbox can go from a deep-sleep state to a high-power state only through these interrupts, i.e., only after the interaction with the patient or the RTC activation during intake time or medication schedule update. Thus, the deep-sleep state enables longer power autonomy of the portable device.

#### 2.2.2. Pillbox Operation

The proposed IoMT platform aims at providing an efficient framework for the improvement of medication adherence. The microcontroller inside the pillbox operates under a low-power state that is interrupted by a DS3231 RTC, which provides two programmable alarms. The first alarm interrupts the microcontroller at a preselected frequency to send temperature, humidity, and adherence data and update the medication schedule according to the database records. The second alarm is programmed to interrupt the microcontroller at the time of the medication intake. The microcontroller, after receiving the updated medication schedule, detects the closest dose and sets the time of the next interrupt accordingly. The frequency of medication schedule updates can be set to allow sufficiently frequent changes (i.e., one-hour intervals), while keeping the energy consumption for the required Wi-Fi connection with the server as low as possible. The repetitive programming of these RTC alarms enables the microcontroller’s continuous alternation between low-power (deep sleep) and high-power states. At the time of medication intake, the microcontroller notifies the patient with the buzzer sound and then goes to deep sleep again. Following patient’s interaction, the deep-sleep state is interrupted and the device indicates the pills that should be received in the current time slot (i.e., 30 min) through display instructions and LED notification, while storing the intake time.

#### 2.2.3. Food-Drug and Supplement-Drug Interaction

Using the pillbox-integrated camera, patients can take photos of the food or supplement they are going to consume to detect possible interactions with their scheduled medication. Through appropriate interaction with the pillbox, the low-power state is interrupted and the patient is guided with display messages to select between the food or supplement option and take a photo using the camera placed on the rear side of the pillbox. The acquired photo is uploaded onto the remote server and the API performs an image analysis [31] to identify possible interactions of the scheduled medication with the food or supplement (Section 2.3.3).

### 2.3. Medication Services Space

The remote server is responsible for the communication between the pillbox, database, web application, and APIs. Through the web application, physicians can insert the patient’s medication schedule and identify crucial drug–drug interactions. If the identified interactions are accepted, the server inserts the updated schedule in the database. Moreover, periodically—at a preselected frequency set for renewal—the pillbox receives the latest medication schedule and sends sensor measurements to the server. For the web server and database operation, the XAMPP platform was employed.

#### 2.3.1. Medication Schedule Insert

Physicians have remote secure access to the server and database data through the web application. Physicians have to fill in patient’s information, the medicine that will be included in the medication schedule, the duration of the treatment, the time of the first dose, and the dose frequency (Supplementary Figure S2). The web application integrates a scheduling algorithm that allows the physicians to complete the medication schedule insert in a way similar to the instructions they provide to the patient, focusing on important therapy parameters, like the duration and dose frequency. The algorithm creates a JSON that appropriately formats the prescription to fit the database structure, where the corresponding doses’ intake time has been calculated.

The web application notifies the physician about possible drug–drug interactions using information from the National Medicine Library Interaction API [27,28,29,30]. When the physician submits the web application form, the remote server makes an API call to check for interactions of the inserted medicine with each of the already prescribed medicines on the same day and notifies the physician if serious interactions are detected (Supplementary Figure S3). This workflow protects patients from harmful drug–drug interactions at the time of visit to their physician by enabling immediate modification of the prescribed medicine to safe alternatives. Through the web application, the physician is also able to remotely monitor the exact intake time for each pill, along with the temperature and humidity measurements. A MariaDB database (Supplementary Figure S4) holds the information about all patients (patient’s name and a unique ID that binds with each pillbox) and their medication schedules separated in two tables (one includes the dates of treatment and the other the dose repetitions during each day). Moreover, it contains temperature and humidity measurements obtained from the pillbox’s integrated sensors, the medicines’ unique identifiers used in drug–drug interactions API check, the food–drug and supplement–drug interactions information, logs of adherence to the treatment, and physicians’ web application login credentials.

#### 2.3.2. Pillbox Schedule Receive

The RTC connected with the pillbox’s microcontroller interrupts regularly (every one hour) its low-power state. For medication schedule renewal, the microcontroller makes an HTTP request over TLS for increased security [26] and sends temperature and humidity measurements to the server; the server responds with database info about the latest medication schedule for the corresponding patient. Secure communication strengthens patients’ data protection and is essential for IoT healthcare devices [34].

#### 2.3.3. Interaction Detection with Pillbox Camera

When patients are about to consume a specific food or a new supplement, they can check for possible interactions with their scheduled medication by taking photos as described in Section 2.2.3. The photo is then sent to the Google Cloud Vision API for recognition. The server, through the database, finds all of the patient’s approaching doses (i.e., within the next two hours) and their medicines. Then it searches the interactions table in the database to find all the interaction keywords for these medicines. Finally, it compares the API response with the interaction keywords. If a keyword match is found, the intake time in the database is postponed (i.e., 2 h later than the current time), so that the undesirable interaction is avoided [19]. Although photos taken from the pillbox’s integrated camera are rather low resolution to achieve fast transmission to the server and reduce microcontroller processing requirements, they are adequately recognized by the API. Vision API can analyze an image and extract labels, which are words that describe its context [35], or text with optical character recognition (OCR) techniques [36]. During food–drug interaction detection, the algorithm extract labels that include the name of the food or drink that will be consumed. Labels extraction cannot identify a pill bottle, which can be the same for many different supplements. Thus, the detection algorithm extracts the text written on the pill bottle and can identify its ingredient from a supplement photo.

### 2.4. Evaluation Study

For the preliminary assessment of the proposed platform, we designed and conducted a two-part experimental procedure. The first part aims to provide an estimation regarding pillbox power consumption during everyday use. The second part aims to evaluate potential medication adherence improvement and usability of the IoMT pillbox, through comparison with a dummy pillbox.

#### 2.4.1. Power Consumption

To evaluate the IoMT pillbox’s portability, we simulated different scenarios that require high-power pillbox states and measured the energy consumption. For this purpose, we used the INA219 current sensor and an Arduino Uno to log the measurements. We performed four experiments that simulated different pillbox working scenarios: (a) pillbox connection to the server for medication schedule update, (b) pillbox sound indication for patient notification at the pill intake time, (c) patient interaction with the pillbox for pill intake, where each pill to be received is indicated, and (d) patient interaction with the pillbox to take a food or supplement photo that is sent to the server for interaction check. Apart from these high-power states, the device operates in the deep sleep state that is characterized by low current. During the low-power state, the current was measured through a multimeter, since INA219 low-current measurements are not accurate.

#### 2.4.2. Medication Adherence and User Acceptability

To evaluate the IoMT pillbox’s potential for improving medication adherence and user acceptance, experiments involving the use of a prototype of the proposed IoMT pillbox, as well as a dummy pillbox, were conducted. The dummy pillbox was visually identical to the IoMT pillbox. A total of 14 adult participants were recruited, signed an informed consent statement, and used both devices under a dense medication schedule to simulate actual treatment plans. The medication schedule contained multiple pill intakes during the day and four remote adjustments. Participants’ ages were 36.6 ± 11.8 years (min: 26, max: 66) and half of them reported to receive medicines or supplements daily.

Each participant was involved in two trials consisting of 12 h of pillbox use each on two separate days. An identical dense pill intake schedule was simulated in both trials, consisting of 10 distinct pills on six occasions (three doses with one pill each, two doses with two pills and one dose with three pills). For the purpose of the trials, actual pills were replaced with candies, which were not consumed during the process. The compartments of both pillboxes were numbered identically (Supplementary Figure S5). In both trials, the participants received printed schedules, a form to monitor pill intake and their activity in an hourly basis, and instructions. During the trial, four adjustments (Table 1) on the given schedule were sent to the participants 90 min before the allocated time of the scheduled change. These adjustments simulated pharmacologically equivalent scenarios that can occur during a patient’s daily routine and lead to treatment failure. In the case of the dummy pillbox trial, adjustments were sent with text messages, while in the case of the IoMT pillbox trial, through changes on the remote server’s database. The remote schedule adjustments shown in Table 1 are generated through a randomization algorithm, which ensures that each distinct type of adjustment takes place once in each trial. After the completion of the experiments for all participants, the total errors and delays in medicine intakes were calculated for each participant and each trial. For the evaluation process, the IoMT pillbox was programmed to skip the dose if the patient tried to receive the pill more than 30 min after the scheduled time, to avoid conflicts between frequent doses and scheduled changes. These cases were reported as medicine intake errors for the IoMT pillbox trial.

After the completion of both trials, participants were asked to provide demographic information and information relevant to user acceptance. To this end, the system usability scale (SUS) [41] was employed, along with seven additional questions (Supplementary Figure S6) and three open-ended questions (best, worst features of the device, and additional suggestions made by the participants). SUS is a 10-question test where every participant provides answers to a 1–5 Likert scale from strongly disagree to strongly agree [42]. Every answer is scored differently if it is odd or even and the total score is calculated. The total score is multiplied by 2.5, so the final score is in a 0–100 scale [43]. SUS provides an easy way to estimate the usability of a product, where a score more than 68 is considered above average [44] and one higher than 80 demonstrates excellent acceptance [45]. According to [46], SUS that is provided to non-native English speakers can cause reliability issues, so we provided the SUS version in the native language of the participants [41].

## 3. Results

### 3.1. Power Consumption Measurements

**Figure 3:**
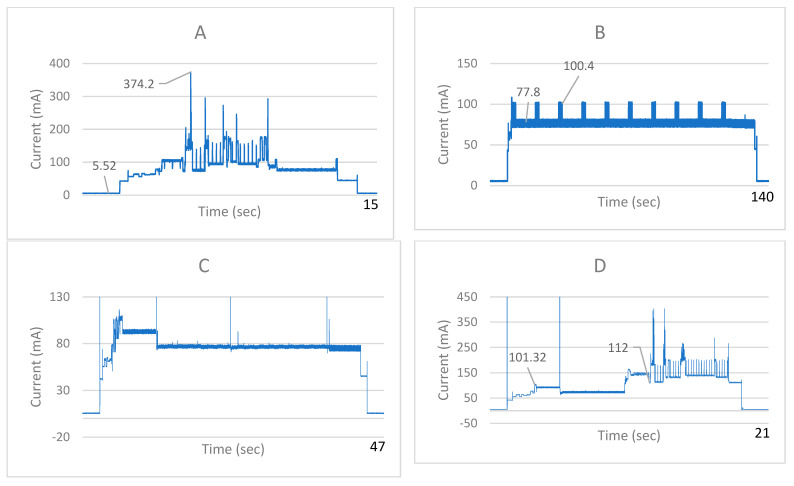
Current measurements with INA219 (battery was at ~3.64 V) as described in Section 2.4.1. (**A**) Medication schedule update, (**B**) sound notifications at the pill intake time, (**C**) LED notifications indicating the appropriate pills for intake, and (**D**) acquisition of food or supplement photo and upload to the server for interaction check.

During the high-power simulations that were described in Section 2.4.1, the battery was at ~3.64 V (Figure 3). The current at low-power state was ~6 mA. Cases A and D include Wi-Fi connection during medication schedule update (case A) and after food or supplement photo for the upload to the server during the interaction check (case D). The results demonstrate that the Wi-Fi connection observed during high current peaks (374.2 mA and >350 mA at cases A and D, respectively) is the most power-demanding process; thus, Wi-Fi connections should be kept at a minimum during device operation. During sound notifications at pill intake time (case B), the current intensity is at 77–100 mA and at ~80 mA during LED notifications indicating the appropriate pills (case C). Cases B and C require pillbox high-power operation but the consumed power is lower as compared to Wi-Fi connectivity demands (cases A, D). The high spikes (>130 mA and >450 mA in cases C and D, respectively) correspond to user interactions with the pillbox (button presses) to activate the indication of each intake pill or the process to take a photo of food or supplement, and they are not actual current measurements. After the initial button press in both cases C and D, the microcontroller leaves the low-power state, which is expected because of the device design, as the buttons operate as interrupts (Figure 2B). Similarly, in cases A and B, the device goes to a high-power state, after the RTC alarms interrupt (Section 2.2.2). Thus, before and right after all these high-power processes (cases A–D), the pillbox reverts to low-power operation at ~6 mA.

### 3.2. Medication Adherence and User Acceptability Results

#### 3.2.1. System Usability Scale

**Figure 4:**
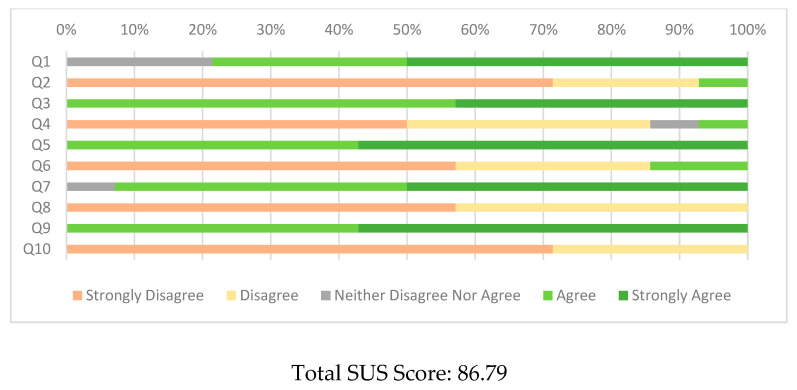
System Usability Scale Answers.

The pillbox was reported as highly acceptable by the participants, with an average score of 86.79. All the participants felt very confident using the device and agreed that the device was easy to use and the functions were well-integrated. The majority of the participants (79%) agreed that they would use the device frequently with the rest (21%) being neutral. Only one participant found the device unnecessarily complex, and no one found it cumbersome to use or requiring excessive training before using it (Figure 4).

#### 3.2.2. Additional Likert and Open-Ended Questions

From the additional questions concerning the experience using the device, 36% of the participants agreed that it was easy to carry the device, 36% neither agreed or disagreed, and the rest disagreed. The majority of the participants (64%) agreed that it was easier to take the medicines from the device compared to the normal packaging (blister), while 29% were neutral and 7% disagreed. All the participants found it beneficial to use the device. Moreover, they found both the indication of the correct medicine at the correct time and amount and receiving notifications helpful. Finally, 86% of the participants found it difficult to remember the right time to take their medicine and thought it is useful to carry the device with them.

From the open-ended questions, positive feedback was received for the LED and sound indications during intake time, along with the instructions appearing on the display. Moreover, the foldable design, the portability, and the consistency of the medicine notifications were considered positive features. On the other hand, most of the participants found the device bulky and suggested its size be adjusted according to the number of pills that one should take. Negative feedback was received for the intensity of sound notifications, which was considered low, the unfolded structure of the pillbox when the lids are open, which was considered unstable, and the low safety of pills storage inside the containers (prone to falling or taken by a child). The participants suggested extra features that could be integrated in a future version of the platform concerning safety and other improvements in the pillbox design, accessibility for patients with disabilities, increased customization, and interoperability.

#### 3.2.3. Medication Adherence Results

In Table 2, the average and standard deviation of the total errors and sum of delays across participants using the dummy and the IoMT pillbox are shown. Data analysis indicated no statistically significant difference (*p* = 0.57) for total errors between dummy and IoMT pillbox use. Comparison of delays in pill intake between the two trials (excluding cases not monitored by the IoMT pillbox—delays more than 30 min per dose) showed that delays related with IoMT pillbox use were lower (*p* = 0.03) than those related with the use of the dummy pillbox. As shown in Table 2, actual reported delays (including more than 30 min per dose) for the dummy pillbox trials were in reality far lengthier, with possible implications in actual treatment.

## 4. Discussion

Results from the power consumption validation process demonstrate that the IoMT pillbox returns in a low-power state, switching automatically from the high-power states imposed by updates or user interaction. This feature, combined with the device’s small size because of its unique foldable design, improves portability and enables indoor and outdoor use without interruptions, contributing in the improvement of medication adherence and the monitoring of pill storage conditions, through temperature and humidity sensors. Moreover, through the pillbox LED strip, every pill combination can be implemented after each schedule update, providing continuous and remote pharmacotherapy adjustment. The observed low-power consumption measurements demonstrate a power efficient hardware design that can increase compliance in long-term use [21]. As demonstrated by all current charts in Figure 3, due to RTC alarms manipulations, the device does not remain inactive until the medication schedule update time. Instead, it goes in deep-sleep mode waiting for the RTC alarm or a user interaction. After the update, another RTC alarm is set at the next intake time, thus enabling continuous low-power operation at ~6 mA. With the 18650 Li-ion battery of 3400 mAh capacity that is included in the pillbox, the device can operate in low-power mode (~6mA) for 566 h and 40 min before it needs recharging [47]. The actual operation time is determined by the frequency of the medication schedule updates that is set, the amount of pill intakes for each patient, and the frequency of food–drug and supplement–drug interactions check.

Results from the comparative evaluation of the dummy and the IoMT pillbox demonstrate statistically significant longer intake delays during the use of the dummy pillbox by the participants. Medication adherence was evaluated under a complex regimen requiring multiple doses and pill combinations throughout the day, with the collected data suggesting that the IoMT pillbox decreases delays between recommended and actual medicine intake time, preventing potential treatment failures. Four different remote dose adjustments (Table 1) were integrated throughout each trial day, representing meaningful and pharmacologically relevant scenarios in real treatment conditions. The IoMT pillbox was able to perform remote treatment adjustments without any action required by the user. This is advantageous in comparison with other state-of-the-art smart pillboxes with pre-defined doses of multiple medicine combinations. The incorporation of this feature in a small and portable device can lead to improved user experience and medication adherence. This is evident in the results of the user acceptance study, denoting that the device is well-accepted by the participants and can be easily integrated in their everyday routine. Moreover, the remote adjustment feature combined with the pillbox’s integrated camera allows collection of data that can lead to the detection and prevention of food–drug and supplement–drug interactions. The developed platform takes advantage of IoMT capabilities to ensure interoperability between high-end image recognition services and low-cost client devices as ESP32-CAM, thus providing a feasible way to use photos provided by the patient to identify and avoid crucial interactions, by instantly altering the medication schedule. The employed cloud services reduce the total platform cost, as the IoMT platform server is not required to handle complex computer vision tasks but only rather low computational demanding services, such as the web application and the database management. Furthermore, the platform supports remote treatment adjustment, enabling the interaction with other medical devices that can provide real-time health indications.

A potential limitation of the proposed framework is the need for a patient’s interaction with the pillbox to record a dose intake. This approach was chosen in an attempt to reduce electronic component complexity that would lead to increased costs and pillbox size. However, the proposed solution allows monitoring of the exact time of pill intake, providing accurate medication adherence data. Extensive device testing is needed during camera usage to assess potential limitations regarding processing by the Google Cloud Vision API or the matching between extracted labels and text with the interaction database keywords. According to participants’ feedback on pillbox limitations, future improvements can include reduced device size depending on the number of pills included in the schedule and enhanced overall device stability.

The use of photos taken through the pillbox-integrated camera provides the ability to identify and avoid harmful interactions in the patient’s daily routine that can reduce pharmacotherapy effectiveness. In addition, photos acquired with the pillbox-integrated camera can be used to address other health-related issues, such as patients’ food habits that can lead to obesity. Moreover, information on food–drug and supplement–drug interactions can be extended to include large-scale cloud databases that are updated with the most recent health data available worldwide. On the other hand, IoMT platform users can provide data on unreported interactions back to these databases to enrich their information. Adjustments concerning the microcontroller peripherals can lead to further improvement of power consumption during the deep-sleep state. The modular platform design enables easy addition of more pillbox sensors or extra devices and wearables. Examples include the addition of a smart watch that could supply heartrate data or the integration of a small microphone in the pillbox that could be used by the patients to enter side effects from medication, thus providing an easy and fast way to gather valuable information and improve pharmacovigilance. Furthermore, remote patient monitoring can provide important data concerning critical health conditions [48]. Additionally, the integration of light and vibration indications during intake time for patients with impaired hearing, or the integration of the Braille system or sound indications for patients with impaired vision, can lead to an IoMT pillbox design suitable for people with disabilities. Moreover, safety improvements such as a lock on the device’s pill compartments restricting access to unauthorized users, smaller device size, automatic pill removal check, and additional features, such as mobile app integration, could further improve user experience.

## 5. Conclusions

In this paper, an IoMT platform featuring a small-sized 3D-printed pillbox, a remote server and database, a web application, and third-party APIs was proposed. The platform aims at enhancing medication adherence by providing extra functionalities that expand personalized medicine concepts. Patients can participate in their pharmacotherapy by using the pillbox-integrated camera to take photos of the food or supplements they are about to consume and if a crucial interaction with their medication is detected, instant changes to their medicine schedule are applied to avoid it. The hardware architecture of the device enables low-power operation and increased portability. A preliminary study with 14 participants that included a complex medication regimen and remote dose adjustments simulating pharmacological equivalent scenarios demonstrated that the use of the proposed IoMT pillbox can result in a statistically significant decrease in medicine intake delays and potentially improve medication adherence.

## Figures and Tables

**Table 1:** Medication Schedule Adjustments.

Medication Adjustments Used in the Study	Pharmacological Equivalent Scenario	Example
Remove medicine from the intake schedule	Biomarkers indicate danger with the usual medication intake schedule	Low blood sugar suggests skipping of the diabetes medication
Change of medicine intake time	Interaction between food and drug	Ciprofloxacin and yogurt simultaneous intake should be avoided [37]
Add medicine: (a) Add extra medicine	Appearance of transient symptoms	An injury may require painkiller temporarily
(b) Double scheduled medicine dose	Biomarkers indicate the need for higher dose than usual	Blood pressure measurements suggest the duplication of the dose
Switch two medicines	Some food-drug interactions should be avoided, while others can be beneficial	Food-Azithromycin capsules interaction should be avoided [38,39], while Food-Cefuroxime Axetil is recommended [40]

**Table 2:** Comparison of Errors and Dose Delays between Dummy and IoMT Pillbox Use.

	Total Errors		Sum of Delays (Minutes)		
	Dummy Pillbox	IoMT Pillbox	Dummy Pillbox (including >30 min delays)	Dummy Pillbox	IoMT Pillbox
Average	1.14	1.43	76.86	25.14	10.29
SD	1.70	1.28	95.34	23.29	8.87
	***p* = 0.57**			***p* = 0.03**	

## Data Availability

Not applicable.

## Supplementary Materials

The following supporting information can be downloaded at https://www.mdpi.com/article/10.3390/s22155818/s1: Figure S1: Pillbox electronic parts on a custom PCB; Figure S2: Web Application where physicians can remotely insert new medication schedules; Figure S3: Web Application alert notifies about drug–drug interactions, after medication form submission; Figure S4: IoMT platform Database design; Figure S5: For the study every pill compartment is numbered for easier pill indication; Figure S6: System Usability Scale and Additional Questions.
