# Supplementary figures and images for: Development of a Low-Power IoMT Portable Pillbox for Medication Adherence Improvement and Remote Treatment Adjustment
Source: Sensors (Basel). 2022 Aug 4;22(15):5818. doi: 10.3390/s22155818 (PMC9370836; doi:10.3390/s22155818)

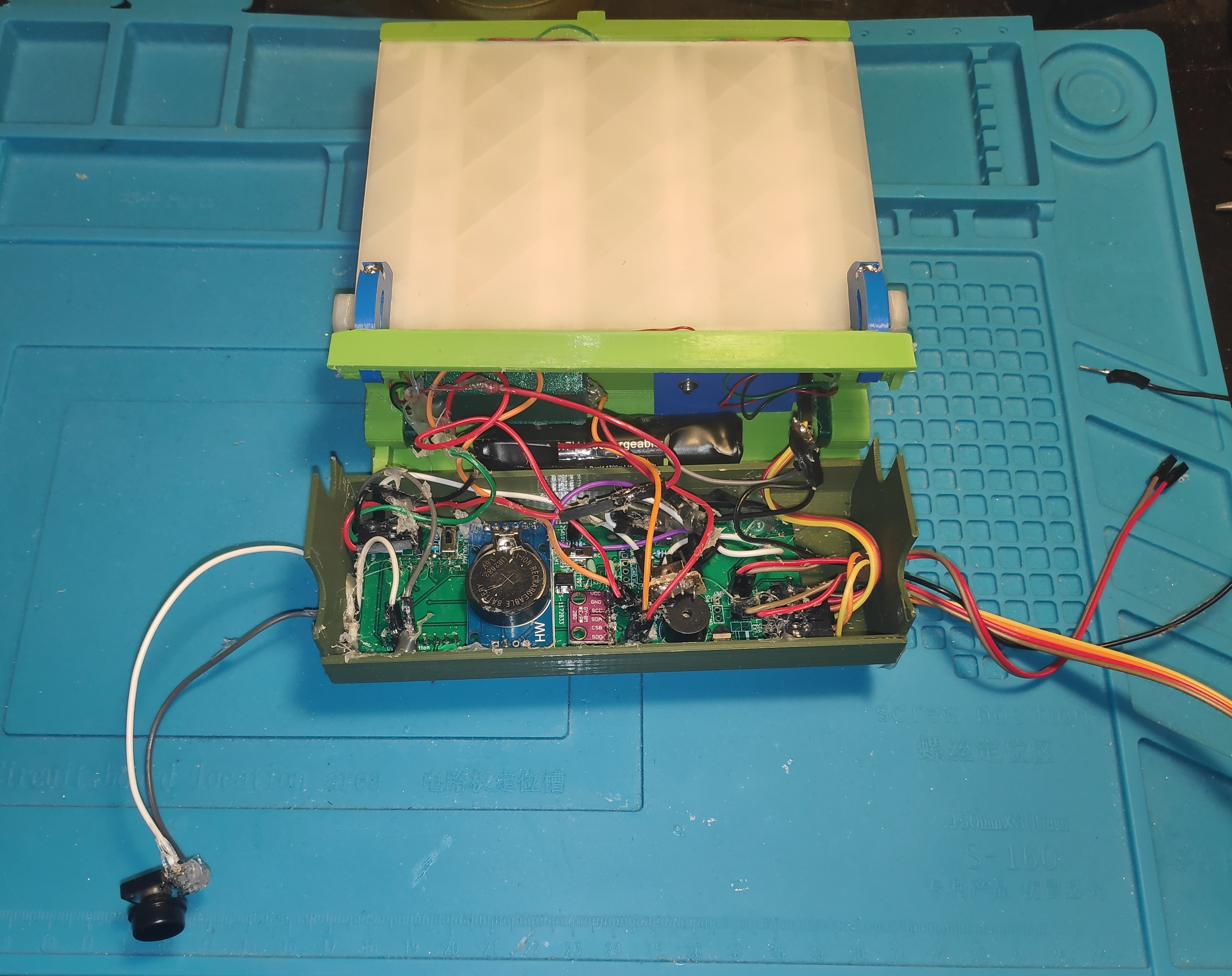

Supplement: Supplementary file 1 [file sensors-22-05818-s001.zip › sensors-1821236-supplementary/S1.jpg]

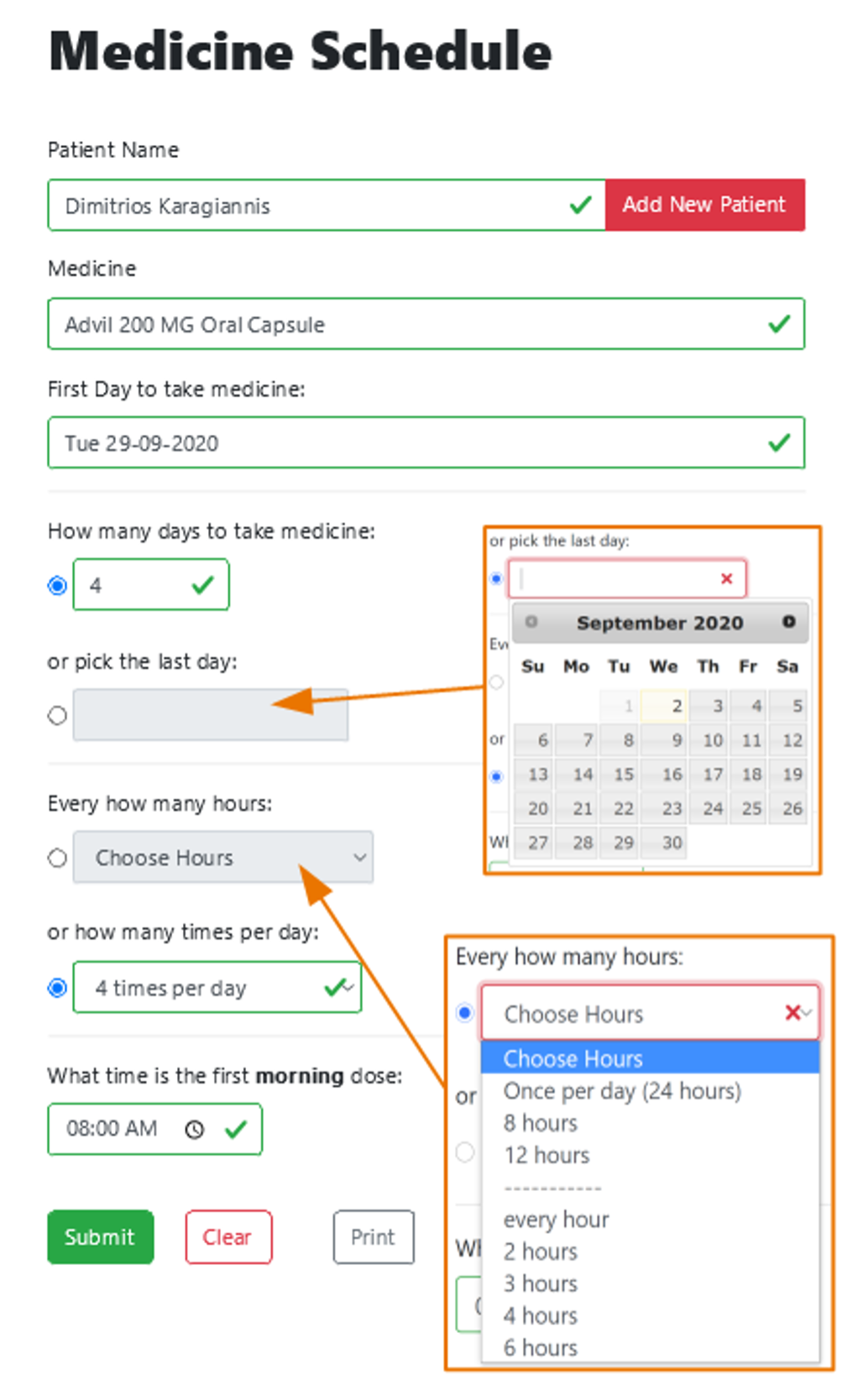

Supplement: Supplementary file 1 [file sensors-22-05818-s001.zip › sensors-1821236-supplementary/S2.png]

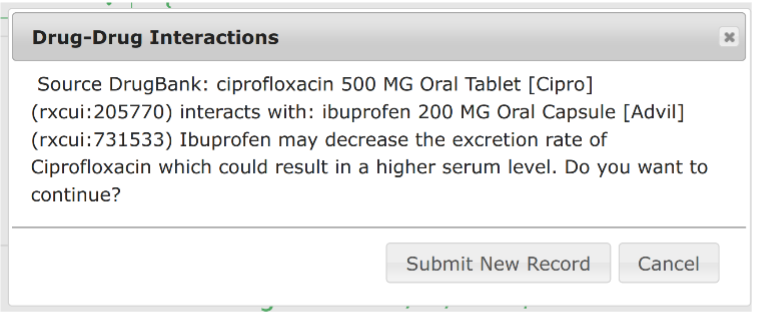

Supplement: Supplementary file 1 [file sensors-22-05818-s001.zip › sensors-1821236-supplementary/S3.png]

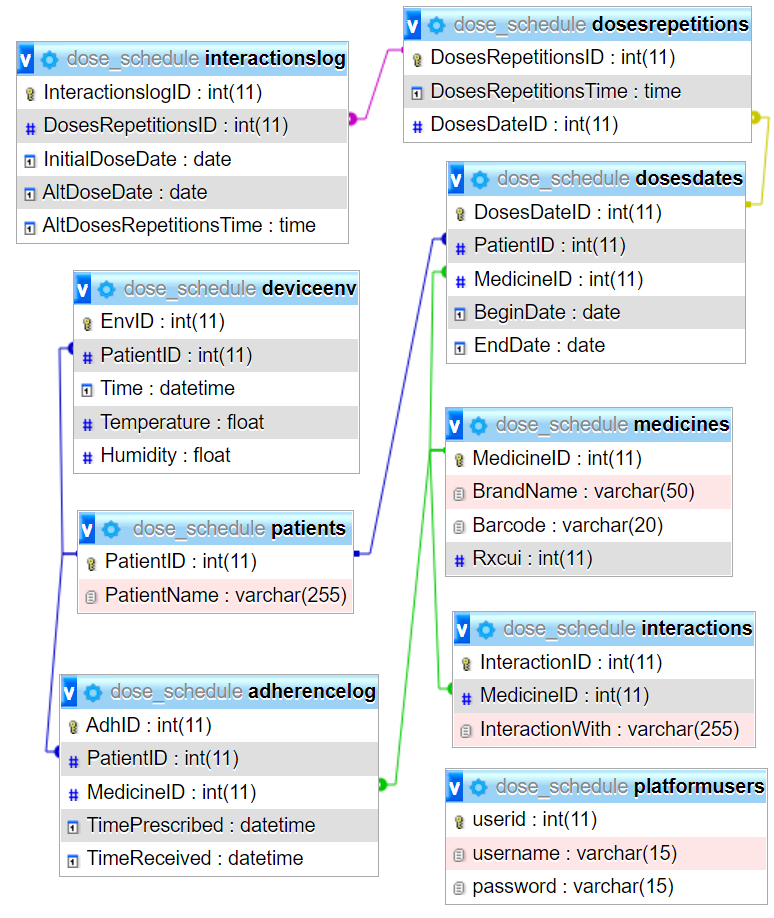

Supplement: Supplementary file 1 [file sensors-22-05818-s001.zip › sensors-1821236-supplementary/S4.png]

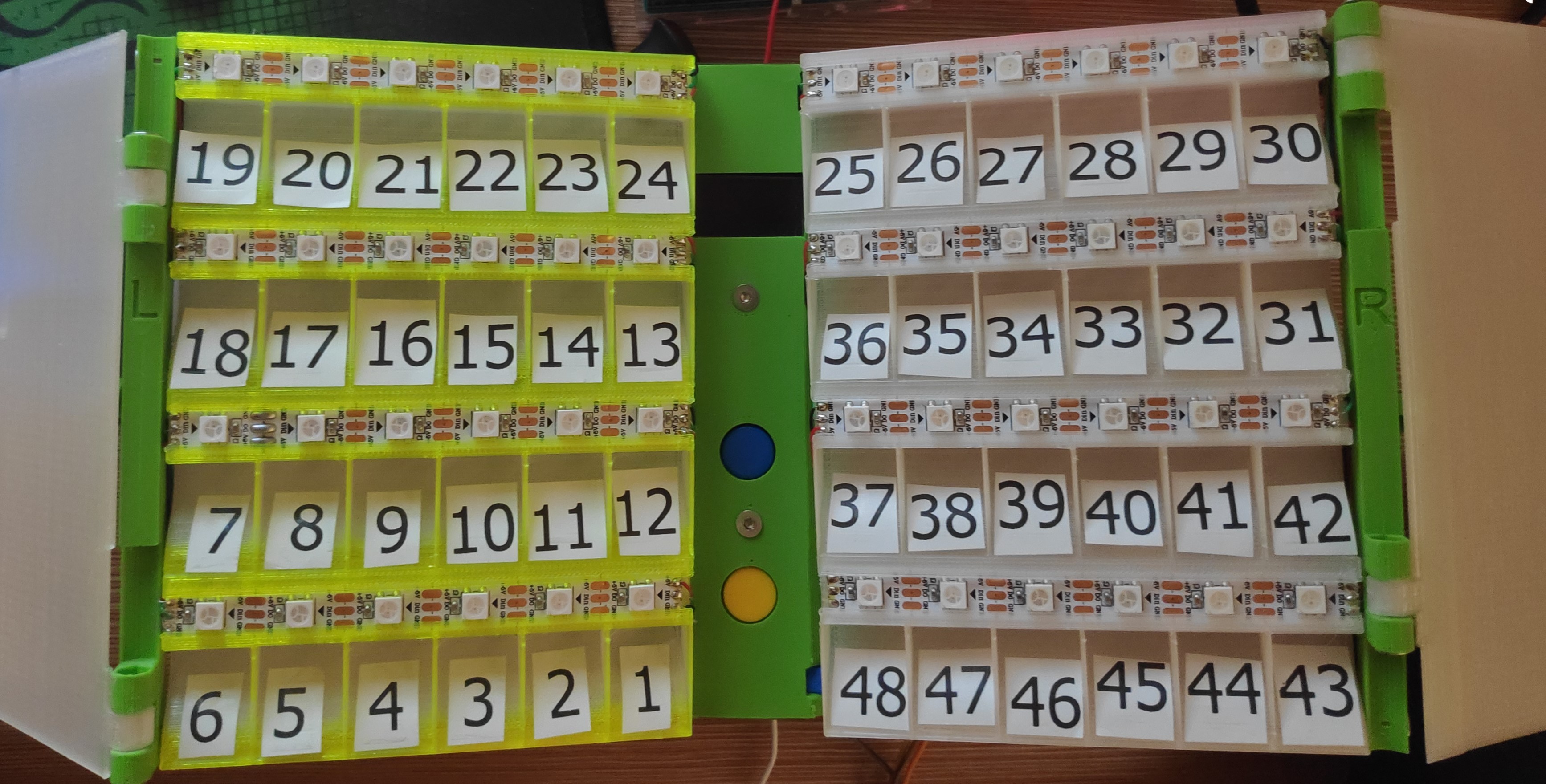

Supplement: Supplementary file 1 [file sensors-22-05818-s001.zip › sensors-1821236-supplementary/S5.jpg]

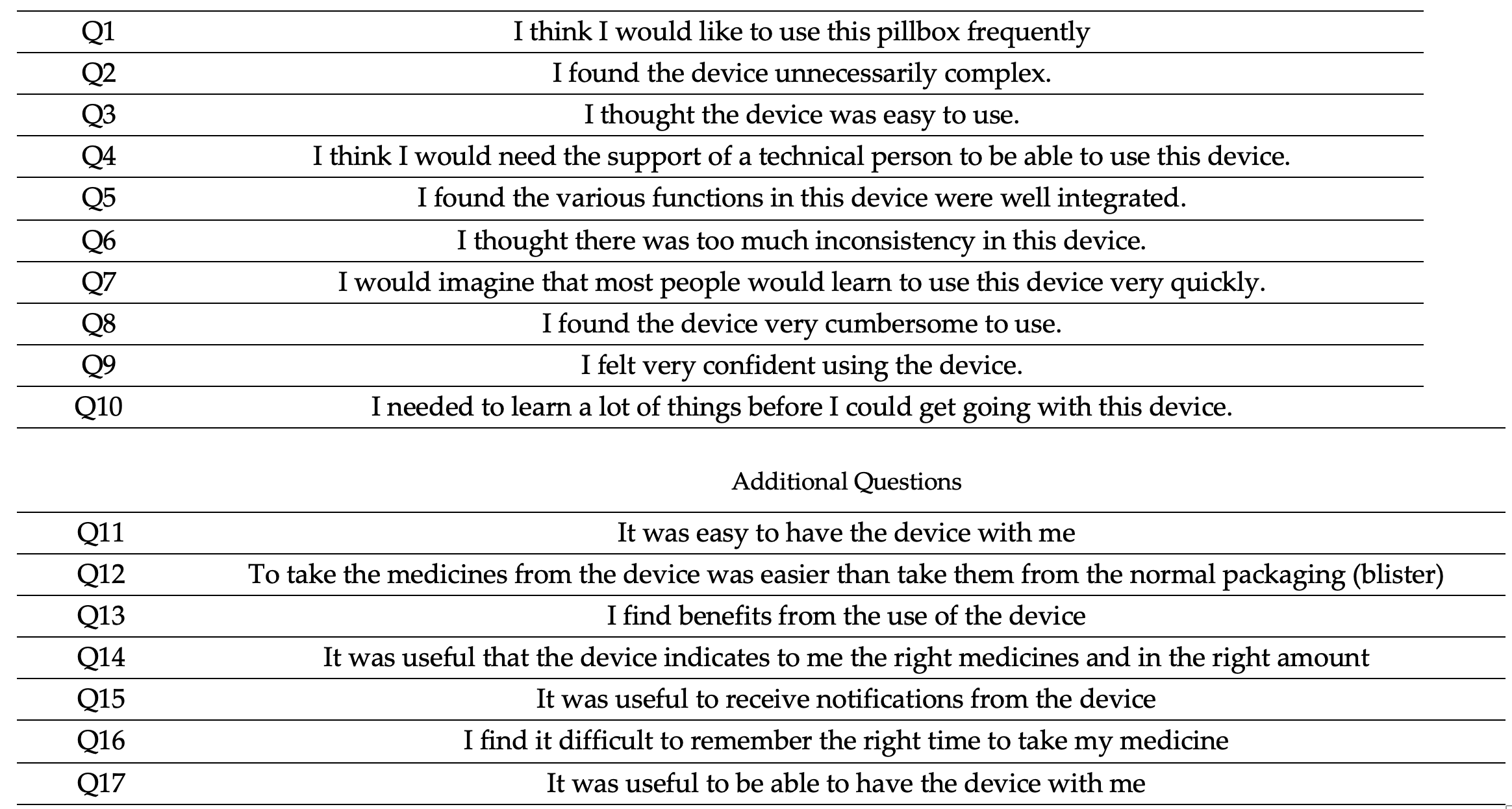

Supplement: Supplementary file 1 [file sensors-22-05818-s001.zip › sensors-1821236-supplementary/S6.png]
